# Distribution Patterns of Ohio Stoneflies, with an Emphasis on Rare and Uncommon Species

**DOI:** 10.1673/031.013.7201

**Published:** 2013-07-15

**Authors:** Scott A. Grubbs, Massimo Pessimo, R. Edward DeWalt

**Affiliations:** 1 Department of Biology and Center for Biodiversity Studies, Western Kentucky University, Bowling Green, Kentucky 42101, USA; 2 University of Illinois, Prairie Research Institute, Illinois Natural History Survey, 1816 S Oak St., Champaign, Illinois 61820, USA

**Keywords:** Midwestern, Nearctic, range reduction

## Abstract

Presently, 102 stonefly species (Plecoptera) have been reported from Ohio. All 9 Nearctic families are represented. Over 90% of the fauna exhibit a combination of broad Nearctic-widespread, eastern Nearctic-widespread, Appalachian, and eastern Nearctic-unglaciated distributions. In contrast, only 2 species display a central Nearctic-Prairie distribution. Seven species of Perlidae are likely no longer present (*Acroneuria evoluta* Klapálek, *A. perplexa* Frison, *Attaneuria ruralis* (Hagen), and *Neoperla mainensis* Banks) or have experienced marked range reductions (*Acroneuria abnormis* (Newman), *A. frisoni* Stark and Brown, and *A. filicis* Frison). Another nearly 31% of the fauna (32 species) are rare, uncommon, or have highly-limited distributions within the state. Twelve of these species have Appalachian distributions, and an additional 8 have eastern Nearctic-unglaciated distributions. The distributional status for each of the 32 rare/uncommon species is discussed.

## Introduction

Prior to DeWalt et al. (2012), the Ohio stonefly (Plecoptera) fauna had been addressed mostly in piecemeal fashion. Walker (1947), Gaufin (1956), Tkac and Foote (1978), Robertson (1979, 1984), Beckett (1987) and Fishbeck (1987) each focused their work mainly at small regional scales. Gaufin's (1956) paper was based on specimens he collected in southern Ohio from 1950–1953. He collected additional material that he took with him to the University of Utah when he joined the faculty in 1953. Later, Gaufin's Ohio stonefly collection was transferred to the Brigham Young University (BYU) after Dr. Richard Baumann moved to BYU in 1975. In the early 1980's Shawn Clark started Ph.D. work at Ohio State University (OSU). Although his dissertation was based in part on chrysomelid beetles, he was encouraged by Dr. Baumann to collect stoneflies when he was visiting lotic systems. By this time, an Ohio stonefly project was established, with periodic collecting trips by Dr. Clark after he had graduated from OSU and had accepted a position with the West Virginia Department of Agriculture in Charleston, West Virginia.

Also during the 1980's, Fred Kirchner of the U.S. Army Corps of Engineers in Huntingdon, West Virginia, became interested in the Ohio stonefly project and often collected there either individually or with Dr. Clark. The Clark material came to BYU when he was hired there in 2002. To date, the majority of F. Kirchner's material has remained with him. The whole of the BYU material was included in the DeWalt et al. (2012) treatment. Tkac's (1979) Ph.D research was somewhat more comprehensive, studying the fauna in detail across the northeastern portion of the state. He also provided the first illustrated taxonomic key for Ohio stoneflies, yet his work was unpublished and has remained largely unrecognized.

DeWalt et al. (2012) showed that the Ohio fauna was represented by 102 species in total, and drainages historically covered by upland deciduous forest and mixed coniferous forests supported the highest species richness. Thirteen species were reported from the state for the first time, but there was also ample evidence that several species of Perlidae likely were no longer present. Although DeWalt et al. (2012) briefly discussed 10 rare/uncommon species within a broad analysis of diversity patterns, there are several more that are similarly uncommon, rare, or display limited distributional ranges within the state. The intent of this paper is to extend upon DeWalt et al. (2012), focusing mainly on species characterized as rare/uncommon.

## Materials and Methods

Freshly-collected specimens and museum material obtained from 19 institutions (Table 1) were used in this study. Data were also acquired from reliable literature sources. Fresh adult specimens were collected with beating sheets, sweep nets, by hand-picking from rocks, tree trunks, and bridges, and through rearing of nymphs. Mature nymphs that could be readily identified to species were also included. Location data for each specimen record were recorded either directly with GPS units or by georeferencing museum label data. Nearctic-scale distribution categories were assigned for all species based on a rapidly-accruing and well-documented literature base of state and Canadian province records (e.g., Stewart and Stark 2002; DeWalt et al. 2012). Distribution categorization has been performed at the state level by Kondratieff and Kirchner (1987, Virginia), Grubbs (1997, Maryland), and Grubbs et al. (2012, Michigan). The previous categories have been modified to include: Appalachian, central Nearctic-Prairie, eastern Nearctic-glaciated, eastern Nearctic-unglaciated , eastern Nearctic-widespread, and Nearctic-widespread.

**Figure 1.**
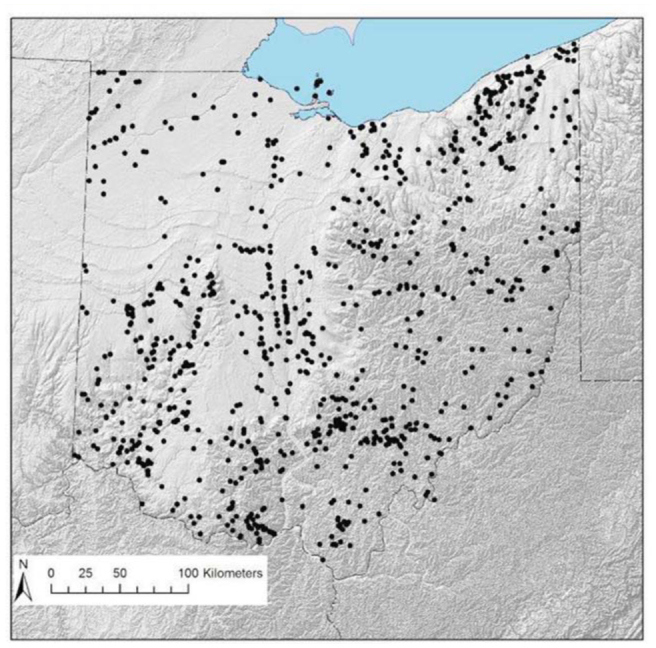
All unique collecting localities for Ohio. High quality figures are available online.

## Results and Discussion

Over 32,000 specimens were accrued for this study, providing records for 102 species (Table 2) from 942 unique localities distributed broadly across the entire state of Ohio, USA (Figure 1). All 9 Nearctic families were represented in Ohio, and the most speciose was Perlidae (34 species). In contrast, Pteronarcyidae was comprised of 2 species of *Pteronarcys*, and *Peltoperla arcuata* Needham was the sole peltoperlid. The pending revision of the eastern Nearctic Isoperlinae (Szczytko and Kondratieff, personal communication) will also likely alter the total number of *Isoperla* known from Ohio. The 2 most commonly collected species were *Allocapnia vivipara* (Claassen) and *Perlesta lagoi* Stark, which were obtained from 223 and 161 separate localities, respectively (Table 2). The only other species reported from > 100 localities was *Acroneuria frisoni* Stark and Brown.

There were several species whose taxonomic status is currently in question. Five Appalachian-distributed species, *Alloperla neglecta* Frison, *Acroneuria kosztarabi* Kondratieff and Kirchner, *Cultus decisus* (Walker), *Malirekus* pr. *iroquois* Stark and Szczytko, and *Pteronarcys* pr. *biloba* Newman, are discussed individually below. The identities of several species of *Perlesta* either have been or still need to be resolved. The record of *Perlesta shubuta* Stark by DeWalt et al. (2012) now refers to the recently-described *P. ephelida* Grubbs and DeWalt (Grubbs and DeWalt 2012). *Perlesta cinctipes* (Banks) has been reported from Kansas and Nebraska south to Arkansas and east to West Virginia (Stark 2004; DeWalt et al. 2013). Stark (2004) provided the first records from Ohio based solely on males. A large series has since been collected from the same locality (Deer Creek, Ross County, Ohio) as Stark (2004). Males matched nicely with the definition of *P. cinctipes*, but the eggs were very similar to *P. decipiens* (Walsh) and distinct from the unique chorionic sculpturing exhibited by eastern Kansas *P. cinctipes* (Stark 1989). *Perlesta lagoi* Stark and *P. nitida* Banks exhibited very similar external genitalic features of males and females, the male aedeagus, and eggs (Grubbs and Stark 2001; Stark 2004). *Perlesta nitida* is typically darkly pigmented, has been reported mainly from the northeastern U.S. (Stark 2004; DeWalt et al. 2013), and was first reported from Ohio by Grubbs and Stark (2001). In contrast, *P. lagoi* is considered to be a southeastern and midwestern U.S. species (DeWalt et al. 2013) and is more lightly pigmented. For this treatment, all specimens were grouped together in the broad sense as *P. lagoi*.

Nearly 80% of the Ohio fauna exhibited a combination of eastern Nearctic-widespread (32 species), Appalachian (25 species), and eastern Nearctic-unglaciated (24 species) distributions (Table 2). In contrast, 6 species were found mainly in the eastern Nearcticglaciated landscapes, and only 2 species, *P. cinctipes* (Banks) and *P. xube* Stark and Rhodes, were distributed within the central Nearctic-Prairie region. Species with eastern Nearctic-widespread distributions were well-represented within the families Capniidae (6 of 15 *Allocapnia* species), Nemouridae (all 3 *Amphinemura* and both *Prostoia* species), Taeniopterygidae (3 of 7 species), and Perlidae (12 of 35 species). The Appalachian fauna was represented by each family, especially Chloroperlidae and Leuctridae. Seven of the 8 *Alloperla* species present in Ohio, and both *Sweltsa* species, have Appalachian distributions. Similarly, 3 of the 7 *Leuctra* species plus *Paraleuctra sara* Hanson, were Appalachian-distributed.

Six of the 9 families were comprised by species with eastern Nearctic-unglaciated distributions. Genera particularly wellrepresented were *Allocapnia* (4 species), both *Zealeuctra* species, and the perlids *Acroneuria* (4 of 10 species), *Agnetina* (2 of 3 species), *Neoperla* (3 of 8 species), and *Perlesta* (4 of 8 species). Not including the 7 that are no longer present or the imperiled perlid species noted above, an additional 46 species have been collected at 10 or fewer distinct localities (Table 2). A subset of 32 species is designated herein as rare/uncommon. Twelve of these 32 species have Appalachian distributions, and an additional 8 have eastern Nearcticunglaciated distributions.

**Figure 2.**
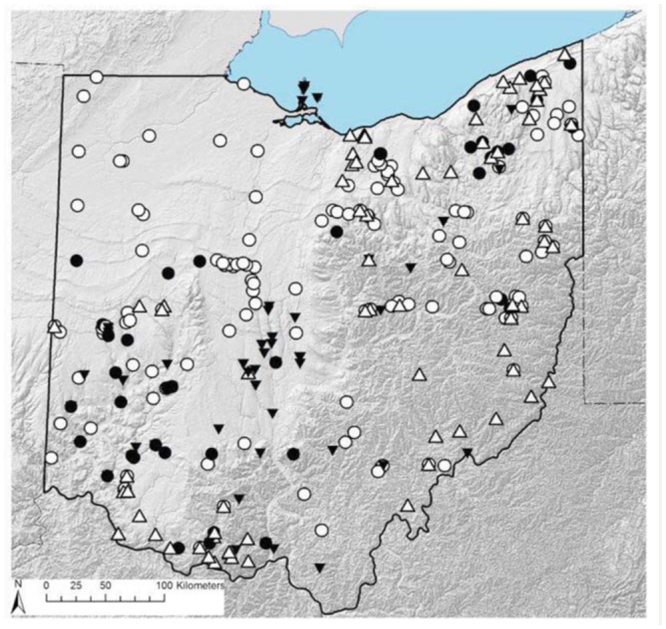
Ohio distribution records of *Acroneuria frisoni* (triangles) and *Perlesta lagoi* (circles) prior to and after 1970. Open symbols: > 1970, filled symbols: < 1970. High quality figures are available online.

Four species of Perlidae, *A. evoluta* Klapálek, *A. perplexa* Frison, *Attaneuria ruralis* (Hagen), and *N. mainensis* Banks, are considered to be no longer present (Table 2; DeWalt et al. 2012). *Acroneuria evoluta*, *A. ruralis* and *N. mainensis* are likewise no longer present in Illinois (DeWalt et al. 2005; DeWalt and Grubbs 2011). *Attaneuria ruralis* similarly is no longer present in Indiana (DeWalt and Grubbs 2011) and Michigan (Grubbs et al. 2012). Three additional perlid species, *A. abnormis* (Newman), *A. filicis* Frison, and *A. frisoni*, have experienced marked range changes since the 1930s (Table 2). *Acroneuria abnormis* inhabits a broad size range of running water systems, and is represented by historical Ohio records from the Hocking River in Athens (1932–1942) and the Ohio River at Ironton (1899) and Marietta (1938). The only recent state records are from northeastern Ohio, namely from upland tributaries to the Mohican River in 1990 and the Grand River in 2006. *Acroneuria filicis* has been recorded mainly from southeastern and south-central Ohio, including several historical series from the Hocking River between 1933 and 1942. Tkac (1979) also collected this species from the Grand River in Lake County. The only recent state record (2008) is from Ohio Brush Creek (Adams County) in southeastern Ohio. *Acroneuria frisoni* has been collected from several more localities than either *A. abnormis or A. filicis*, but is similarly comprised of relatively few recent records (Figure 2), mainly from the unglaciated southern region and from the far northeastern portion of the state. In contrast, *P. lagoi* is still distributed broadly in areas of the state (e.g., northeastern quarter) where *A. frisoni* can no longer be found.

Two Appalachian species whose specific identity needs to be resolved, *Alloperla concolor* Ricker*/neglecta* Frison, and *Cultus verticalis* (*Banks*)/*decisus decisus* (Walker), have rarely been collected in Ohio and may be restricted solely to the northeastern drainages. Most Appalachian-distributed species have been collected mainly in northeastern or south-central Ohio.

## Rare/uncommon species

### Appalachian

#### *Allocapnia frisoni* Ross and Ricker

**Collection records. USA, Ohio:** Athens Co., Rock Riffle, Athens, 39.3196, -82.0764, 5.III.1942, WE Stehr, 2♂ (INHS); Geauga Co., tributary to East Branch Chagrin River, Stebbins Gulch, Holden Arboretum (HA), 41.6130, -81.2656, 26.XII.1975, ♀ (Tkac 1979), same but 26.I.1976, ♂ (Tkac 1979); Hocking Co., tributary Queer Creek, 3 km SSW Cedar Falls, Ash Cave, Hocking Hills State Park (HHSP), 39.3995, -82.5445, 6.III.1938, TH Frison, 6♂, 6♀ (INHS); East Fork, Ash Cave, 39.3954, -82.5473, 24.I.1984, SM Clark and Kammerer, 2♂ (BYU); East Fork Queer Creek, 6.5 km SE South Bloomingville, Hocking State Forest (HSF), 39.3917, -82.5319, 27.II.2011, SA Grubbs, ♂, 2♀ (WKU); Lawrence Co., Caulley Creek, 14 km SSE Oak Hill, Wayne National Forest (WNF) 38.7672, -82.5448, 26.II.2011, SA Grubbs, ♂, ♀ (WKU); Ross Co., Piny Run, 17 km ENE Chillicothe, Tar Hollow State Forest, 39.3685, -82.7840, 19.II.2011, SA Grubbs, ♂ (WKU).

**Remarks.** This is mainly an Appalachian-distributed species, known from New York southwest to Kentucky but also with isolated records from Wisconsin (Ross and Ricker 1971; DeWalt et al. 2013). There are now several valid records for Ohio, mainly in the northeastern and south-central counties (Figure 2).

#### *Leuctra alexanderi* Hanson

**Collection records. USA, Ohio:** Belmont Co., tributary to Belmont Lake, Barkcamp State Park, 40.0572, -81.0386, 3.VI.1989, RW Baumann and RF Kirchner, 3♂, 4♀ (BYU); Portage Co., spring, West Branch State Park (WBSP), 41.1287, -81.1460, 5.VI.1992, BA Foote, 12♂, 14♀ (BYU); Porter Road spring, WBSP, 41.1134, -81.1221, 1.VI.1978, ♀, ♂ (Tkac 1979); same but 9.VI.1978, 19♀, 19♂ (Tkac 1979); same but 15.V–15.VI.1979, R Hunt, 24♀, 32♂ (BYU).

**Remarks.** This species is known from Pennsylvania southwest to central Kentucky (DeWalt et al. 2013), with only 3 known state localities in northeastern Ohio (Figure 2).

#### *Leuctra duplicata* Claassen

**Collection records. USA, Ohio:** Ashtabula Co., Crooked Creek, Callahan Rd., 41.6425, 80.9718, 2.VI.1989, RW Baumann and RF Kirchner, 2♂ (BYU); same but 3.VI.1997, RW Baumann and BC Kondratieff, 7♂, 9♀ (BYU); spring-fed tributary to Crooked Creek, Callahan Rd., 41.6425, -80.9737, 2.VI.1989, RW Baumann and RF Kirchner, 42♂, 28♀ (BYU).

**Remarks.** This species has recorded from the Canadian Maritime Provinces south to Virginia (DeWalt et al. 2013), known only from 2 adjacent localities in far northeastern Ohio (Figure 2).

**Figure 3–6.**
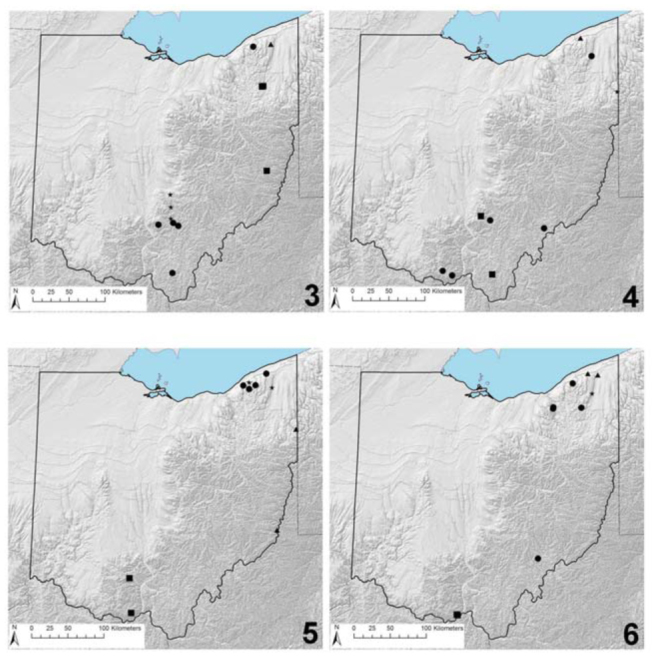
Ohio distribution records. 3. *Allocapnia frisoni* (circles), *Leuctra alexanderi* (squares), *L. duplicata* (triangles), and *L. tenella* (stars). 4. *Acroneuria kosztarabi* (circles), *Alloperla idei* (squares), *A. concolor/neglecta* (triangles), and *Sweltsa lateralis* (stars). 5. *Cultus verticalis/decisus decisus* (circles), *Isoperla holochlora* (squares), *Malerikus* cf. *iroquois* (triangles), and *Pteronarcys* cf. *biloba* (stars). 6. *Allocapnia illinoensis* (circles), *A. indianae* (squares), *A. pechumani* (triangles), and *A. pygmaea* (stars). High quality figures are available online.

#### *Leuctra tenella* Provancher

**Collection records. USA, Ohio:** Fairfield Co., small stream above Lake Pamona, Barnebey Center, 39.7500, -82.6000, 26.V.1982, SM Clark, 4♂, ♀ (BYU); Clear Creek Metropark, 39.5913, -82.5872, 15.V.1973, NWB, ♂ (RFK); same but 19.V.1973, NWB, ♂, ♀ (RFK); Hocking Co., tributary to Pine Creek, 5 km NNE South Bloomingville, HSF, 39.4468, -82.5850, 26.V.2010, SA Grubbs, 2♂ (WKU).

**Remarks.** This is mainly an Appalachian species, recorded from the Canadian Maritime Provinces west to Minnesota and south to West Virginia (DeWalt et al. 2013). This species is currently known from only 3 localities in south-central Ohio (Figure 2).

#### *Alloperla idei* Ricker

**Collection records. USA, Ohio:** Lawrence Co., Buffalo Creek, 17 km SSE Oak Hill, WNF, 38.7460, -82.5445, 27.V.2010, SA Grubbs, 3♂, 9♀ (WKU); Pickaway Co., tributary to Laurel Run, Laurelville, 39.4739, 82.7426, 23.V.1950, AR Gaufin, 2♂ (BYU).

**Remarks.** This species is distributed from eastern Canada south to Alabama and Mississippi (Surdick 2004; DeWalt et al. 2013). Only 2 state records exist, both in southcentral Ohio (Figure 3).

#### *Alloperla concolor* Ricker*/neglecta* Frison

**Collection records. USA, Ohio:** Lake Co., Paine Creek, Paine Rd, Leroy Township, Paine Falls Metropark, 41.7170, -81.1436, 31.V.1975, ♂ (Tkac 1979).

**Remarks.** *Alloperla neglecta* is a southern Appalachian species, with verified records only from North Carolina, Tennessee, and Virginia. Tkac (1979) indicated that he had collected this species from Paine Creek in far northeastern Ohio (Figure 3). The line drawings of the epiproct of the single male strongly suggested he had obtained either *A. neglecta* or *A. concolor* (Ricker). Because these two species have superficially-similar epiprocts (Kondratieff and Kirchner 1993a; Surdick 2004), fresh specimens are needed to verify which species Tkac had collected since his material could not be located for reexamination. *A. concolor* exhibits a distribution from eastern Canada south to Pennsylvania and West Virginia (Kondratieff and Kirchner 1993a; Surdick 2004; DeWalt et al. 2013).

#### *Sweltsa lateralis* (Banks)

**Collection records. USA, Ohio:** Mahoning Co., Grays Run, Lowellville, 41.0440, 80.5396, ♀ (Fishbeck 1987).

**Remarks.** This species is known from New Brunswick west to Ontario and south to Georgia (Surdick 2004; DeWalt et al. 2013) and is especially abundant in Appalachian headwater streams (e.g., Huryn and Wallace 1987). Fishbeck (1987) presented the sole state record, from a forested headwater stream in northeastern Ohio (Mahoning County; Figure 3). Additional populations are expected to be located in relatively undisturbed headwater catchments in eastern Ohio.

#### *Acroneuria kosztarabi* Kondratieff and Kirchner

**Collection records. USA, Ohio:** Adams Co., Upper Churn Creek, 38.7776, -83.3345, 15.VI.1995, no collector information, ♀ (OBS); Hocking Co., Queer Creek, NE South Bloomingville along Hwy 664, HHSP, 39.4236, -82.5908, 19.VI.1996, H Sharb, ♀ (OBS); Scioto Co., Mackletree Run, 8 km NW Friendship at confluence with Lake Roosevelt, Shawnee State Forest (SSF), 38.7239, 83.1815, 20.VI.1999, EG Chapman, 3♀ (OBS); Trumbull Co., Mill Creek, 10 km NE Middlefield at Sweet West Rd. bridge, 41.4894, -80.9567, 4.VII.1994, V Fazio, ♀ (OBS); Washington Co., Little West Branch, CR 3 bridge, east of Decaturville, 39.3322, 81.7232, 18.VI.1996, T Troutner, ♀ (OBS).

**Remarks.** This species has previously been known only from southwestern Virginia (Kondratieff and Kirchner 1993b). Several series of females from the 4 localities across southern Ohio, plus a fifth series from the northeastern portion of the state (Figure 3), have been identified as *A. kosztarabi*. Males and mature eggs are needed for verification.

#### *Cultus verticalis* (Banks)*/decisus decisus* (Walker)

**Collection records. USA, Ohio:** Geauga Co., tributary to East Branch Chagrin River, Stebbins Gulch, HA, 41.6180, -81.2792, 20.V.1976, nymph (Tkac 1979); Lake Co., Piersons Creek, Kirtland Hills at Sperry Rd., 41.6280, -81.3149, 11.V.1978, 3 nymphs (Tkac 1979); Mill Creek, Doty Rd., 41.7400, - 81.0268, 22.V.1978, ♂, 11 nymphs (Tkac 1979); Penitentiary Glen, Penitentiary Glen Preserve, 41.6130, -81.3417, ♂ (Robertson 1979).

**Remarks.** Tkac (1979) collected *Cultus* and *Diploperla robusta* Stark and Gaufin from several upland streams in northeastern Ohio (Figure 4). His line drawings of the male terminalia clearly show he had collected *Cultus*, but it is near-impossible to delineate whether there were of *C. verticalis* or *C. decisus decisus*. This is especially problematic because the Tkac specimens were collected prior to the Stark et al. (1988) partitioning of *C. decisus sensu lato* into 3 taxonomic units. Both *C. verticalis* and *C. d. decisus* have been collected as recently as the 1990s in western Pennsylvania, suggesting that Tkac (1979) may have obtained either species. Fresh specimens are needed because his material could not be located for study.

#### *Isoperla holochlora* (Klapálek)

**Collection records. USA, Ohio:** Pike Co., creek below Pike Lake, Pike Lake State Park, 8.VI.1989, 39.1538, -83.2146, SW Baumann and SM Clark, ♂ (BYU); Scioto Co., Mackletree Run, 12 km SSW West Portsmouth, SSF, 38.7236, -83.1820, 15.IV.2006, RE DeWalt and SK Ferguson, 2 nymphs (INHS).

**Remarks.** This is an Appalachian endemic known from Quebec and Nova Scotia south to Georgia (DeWalt et al. 2013). This species has been obtained from only 2 localities in southcentral Ohio (Figure 4).

#### *Malirekus* pr. *iroquois* Stark and Szczytko

**Collection records. USA, Ohio:** Mahoning Co., Grays Run, Lowellville, 41.0440, 80.5396, ♂ (Fishbeck 1987); Monroe Co., tributary to Stillhouse Run, 39.7815, 80.8529, 1.IV.2001, M Leuhrs, 2 nymphs (OEPA).

**Remarks.** *Malirekus* is an Appalachian genus comprised of only 2 species, *M. hastatus* (Banks) and *M. iroquois*. Fishbeck (1987) reported that he had collected a single male of *M. hastatus* from a headwater stream in northeastern Ohio (Figure 4). At that time, however, only a single species was recognized before Stark and Szczytko (1988) partitioned a southern taxonomic unit (*M. hastatus*) from a northern unit (*M iroquois*). Presently, *M. hastatus* is known from Georgia north to West Virginia and southeastern Pennsylvania, while *M. iroquois* has been recorded from the Canadian Maritime Provinces south to western Maryland (Stark and Szczytko 1988; Kondratieff 2004; DeWalt et al. 2013). Fishbeck (1987) likely collected *M. iroquois*, but fresh adult material is needed for confirmation. His *Malirekus* specimen was not available for examination. Two additional *Malirekus* nymphs have since been collected from a headwater stream in Monroe County in the far southeastern Ohio.

#### *Pteronarcys* pr. *biloba* Newman

**Collection records. USA, Ohio:** Ashtabula Co., Indian Creek, Montgomery Rd., RM 1.3, 41.5640, -80.9328, 11.IX.2007, nymph (Bolton 2010); Lake Co., Piersons Creek, Kirtland Hills at Sperry Road, 41.6280, -81.3149, 11.V.1978, nymph (Tkac 1979); same but 20.V.1978, nymph (Tkac 1979).

**Remarks.** This species known from the Canadian Maritime Provinces southwest through the southern Appalachian region (Nelson 2000; DeWalt et al. 2013). Tkac (1979) first collected nymphs that strongly resembled *P. biloba* from a small, upland stream in Lake County (Figure 4). Unfortunately, adults were not obtained. Bolton (2010) recently reported that he had collected a *P*. pr. *biloba* nymph from Ashtabula County. Similar to Tkac (1979), however, adults have not been collected for species verification.

### Eastern Nearctic-glaciated

#### *Allocapnia illinoensis* Frison

**Collection records. USA, Ohio:** Cuyahoga Co., tributary to Chagrin River, Brecksville at stone bridge, Brecksville Reservation, 41.3010, -81.6097, 21.III.1977, 17♂, 2♀ (Tkac 1979); Chippewa Creek, Brecksville at bridge, Brecksville Reservation, 41.3171, 81.5931, 21.II.1977, 3♂, ♀ (INHS); Geauga Co., tributary to East Branch Chagrin River, Stebbins Gulch, HA, 41.6180, -81.2792, 24.I.1978, 2♂, ♀ (Tkac 1979); Portage Co., organic seep, 1 mi NW Garrettsville, Hiram College Field Station, 41.3127, -81.1351, 20.XII.1990, RE DeWalt and TS DeWalt, 5♂ (BYU); Washington Co., Coal Run, 0.5 mi W Bartlett, 39.4219, -81.8284, 17.III.1966, PW Smith, ♂ (INHS).

**Remarks.** This species is distributed broadly across the Great Lakes region south to isolated localities in Maryland and Virginia (Grubbs 1997; DeWalt et al. 2013). It has only recently been confirmed from Indiana (DeWalt and Grubbs 2011). The 5 known state records are all from eastern Ohio (Figure 5).

#### *Allocapnia pechumani* Ross and Ricker

**Collection records. USA, Ohio:** Ashtabula Co., Mill Creek, Cork Cold Springs Rd, Harpersfield Township, 41.7240, -80.8632, 18.III.1978, ♀ (Tkac 1979); Lake Co., Mill Creek, Cork Cold Springs Rd, 41.7240, 81.0028, 18.III.1978, ♀ (Tkac 1979); Mill Creek, Doty Rd, 41.7400, -81.0268, 23.II.1976, 24♂, 12♀ (Tkac 1979); same but 18.III.1978, 3♂ (Tkac 1979).

**Remarks.** This species occupies a limited range in the northeastern Nearctic region. Prior to DeWalt et al. (2012), this species had been reported only from New Brunswick, Quebec, New York, and Pennsylvania (Ross and Ricker 1971; DeWalt et al. 2013). Within Ohio, this species is known only from Mill Creek from the far northeastern portion of thestate (Figure 5).

#### *Allocapnia pygmaea* (Burmeister)

**Collection records. USA, Ohio:** Trumbull Co., Mill Creek, 10 km NNE Middlefield at Sweet West Rd Bridge, 41.4890, -80.9657, 7.III.1976, ♂, ♀ (Tkac 1979); same but 18.II.1978, 6♂, 4♀ (Tkac 1979).

**Remarks.** This species is distributed across much of the central and eastern Nearctic region, south to Tennessee, and with an Ozark Mountains disjunction population in central Missouri (Ross and Ricker 1971; DeWalt et al. 2013). The only record for Ohio is from an upland stream in the northeastern portion of the state (Figure 5).

### Eastern Nearctic—unglaciated

#### *Allocapnia indianae* Ricker

**Collection records. USA, Ohio:** Scioto Co., Odell Creek, W Portsmouth Rd 25, 38.7032, 83.1159, 19.III.1950, WE Ricker, 3♂, 2♀ (INHS); Turkey Creek, W of Portsmouth, 38.6970, -83.1003, 19.III.1950, WE Ricker, 12♂, 6♀ (INHS); Turkey Creek, 9 mi E Blue Ck. Rd. 125, 38.7272, -83.1727, 19.III.1950, WE Ricker, ♂, ♀ (INHS).

**Remarks.** This species is known only from central Kentucky north to the unglaciated southern portions of Indiana and Ohio, plus a northern disjunct in New York (Ross and Ricker 1971; DeWalt et al. 2013). The only state records pertain to 3 localities in Scioto County in far south-central Ohio (Figure 5).

#### *Zealeuctra fraxina* Ricker and Ross

**Collection records. USA, Ohio:** Hocking Co., stream, Ash Cave, HHSP, 39.3988, 82.5450, 6.III.1938, TH Frison, ♂ (CNC); East Fork, Ash Cave, 39.3954, -82.5473, 21.III.1975, RW Baumann and OS Flint, 2♀ (BYU); Lawrence Co., tributary to Storms Creek, 12 km SW Waterloo, WNF, 38.6313, 82.5810, 26.II.2010, SA Grubbs, ♀ (WKU).

**Remarks.** This species was described from Ash Cave in the Hocking Hills area in southern Ohio (Ricker and Ross 1969). Surprisingly, there are only 2 additional Ohio records for this species, a second from the Ash Cave area, and 1 taken recently from an upland headwater stream in Wayne National Forest (Figure 6). Sampling efforts during mid- to late winter should reveal additional populations.

#### *Taeniopteryx lita* Frison

**Collection records. USA, Ohio:** Meigs Co., Ohio River, 3 mi S Portland, 38.9358, 81.7571, 12.II.1992, SM Clark, nymph (BYU).

**Remarks.** This is a riverine species distributed across the unglaciated portions of the central and eastern Nearctic region (Stewart 2000; DeWalt et al. 2013). A single state record is available from the Ohio River bordering Meigs County in southeastern Ohio (Figure 6).

#### *Acroneuria covelli* Grubbs and Stark

**Collection records. USA, Ohio:** Athens Co., Hocking River, Athens, 39.3292, -82.1222, 8.VI.1937, WC Stehr, ♀ (INHS); Hocking River, Athens Township, 39.3324, -82.0998, 4.VII.1941, JD Walker, ♀ (CNC), same but 6.VII.1941, JD Walker, ♂ (CNC); New Marshfield, 39.3250, -82.2181, 9.VII.1942, J Herron, ♀ (INHS).

**Remarks.** This is a riverine species, described from the Ohio River along the Indiana-Kentucky border (Grubbs and Stark 2004). Similar to Indiana, verified records from Kentucky and Tennessee are also from large rivers (Grubbs and Stark 2004; Tarter et al. 2006). The only Ohio records are from the Hocking River in the vicinity of Athens (Figure 6). Although these populations are likely no longer present, contemporary series from the mainstem Ohio River are expected.

**Figure 7–10.**
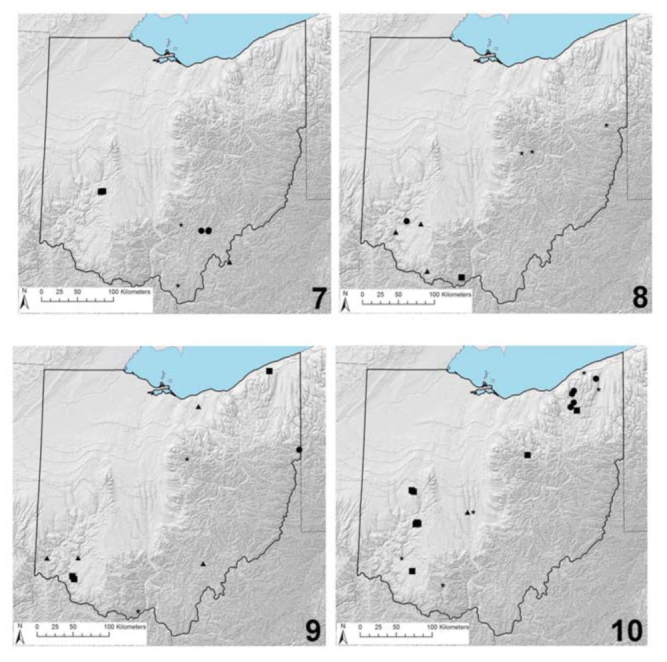
Ohio distribution records. 7. *Acroneuria covelli* (circles), *Agnetina annulipes* (squares), *Taeniopteryx lita* (triangle), and *Zealeuctra fraxina* (stars). 8. *Allocapnia smithi* (circle), *Isoperla burksi* (square), *Neoperla gaufini* (triangles), and *Perlesta golconda* (stars). 9. *Isoperla dicala* (circle), *Neoperla coosa* (squares), *Perlinella drymo* (triangles), and *Prostoia completa* (stars). 10. *Isoperla signata* (circles), *Nemoura trispinosa* (squares), *Pteronarcys dorsata* (triangles), and *Taeniopteryx parvula* (stars). High quality figures are available online.

#### *Agnetina annulipes* (Hagen)

**Collection records. USA, Ohio:** Greene Co., Little Miami River, Clifton, 39.7983, 83.8270, 5.VI.1930, JN Knull, ♂ (OSU); Little Miami River, Clifton Falls, John Bryan State Park (JBSP), 39.7857, -83.8608, 1.VI.1914, DJ Knull and JN Knull, ♂ (OSU); Scioto Co., 1.VI.1925, CH Kennedy, ♂ (OSU).

**Remarks.** This is mainly a coastal plain species, reported from Louisiana east to Florida and north to Indiana and Pennsylvania (Stark 1986; DeWalt et al. 2013). Within Ohio, this species has been obtained only from the Little Miami River in the Clifty Falls area and an additional unspecified locality in Scioto County (Figure 6). This species is similarly known from only the southern tier of neighboring Indiana (Grubbs 2004; DeWalt and Grubbs 2011).

#### *Neoperla gaufini* Stark and Baumann

**Collection records. USA, Ohio:** Brown Co., West Fork Eagle Creek, 3.5 km SW Decatur, 38.7924, -83.7344, 6.VII.2008, RE DeWalt, ♀ (INHS); Clinton Co., Cowan Creek, 39.3840, -83.8608, 5.VII.1951, AR Gaufin, ♂, 3♀ (BYU); Hamilton Co., Little Miami River, Loveland, 39.2686, -84.2605, 28.VI.1953, AR Gaufin, 9♂, 16♀ (BYU); same but 2.VII.1953, AR Gaufin, 5♂, 12♀ (BYU).

**Remarks.** This species is known only from Indiana, Kentucky, and Ohio (Stark 2004; DeWalt et al. 2013). This species is presently known from 3 localities in the unglaciated southwestern portion of Ohio (Figure 7). Additional collecting should reveal several additional populations, particularly from smaller streams that transition to intermittent flow by early summer.

#### *Perlesta golconda* DeWalt and Stark

**Collection records. USA, Ohio:** Columbiana Co., Nancy Run, Franklin Township at Hull Rd., 40.6610, -80.8563, 30.VI.1997, EG Chapman, 4♂ (OBS); Coshocton Co., Mohawk Creek, 0.5 km E Mohawk Village at Co. Rd. 82 bridge, 40.3205, -82.0741, 26.VI.1999, SW Chordas III and J Thompson, 2♂ (OBS); Knox Co., Wakatomika Creek, 4 km NE Bladensburg at Front Royal Rd. bridge, 40.2986, -82.2448, 26.VI.1999, SW Chordas III and J Thompson, ♂ (OBS).

**Remarks.** This is a riverine species known currently from Nebraska east to Indiana (DeWalt et al. 1998; Stark 2004; Grubbs and DeWalt 2008; DeWalt and Grubbs 2011). Museum material from 3 locations in central and eastern Ohio have since been identified (Figure 7).

#### *Isoperla burksi* Frison

**Collection records. USA, Ohio:** Scioto Co., Mackletree Run, 12 km SSW West Portsmouth, SSF, 38.7236, -83.1820, 15.VI.2006, RE DeWalt and SK Ferguson, 3 nymphs (INHS).

**Remarks.** This species has been recorded from a narrow latitudinal band from Oklahoma east to New Jersey and the Carolinas (DeWalt et al. 2013). Within Ohio, this species has been reported from only 1 locality, a small stream in Shawnee State Forest (Figure 7). *I. burksi* is apparently restricted to the unglaciated counties in southern Ohio, similar to its distribution in Illinois and Indiana (DeWalt and Grubbs 2011).

### Eastern Nearctic—widespread

#### *Allocapnia smithi* Ross and Ricker

**Collection records. USA, Ohio:** Warren Co., tributary to Little Miami River, 10 km ESE Lebanon, Fort Ancient State Memorial, 39.4097, -84.0949, 12.II.1966, FJ Moore, ♀ (INHS).

**Remarks.** This species is distributed from central Alabama north through the unglaciated southern portions of Illinois, Indiana, and Ohio (Ross and Ricker 1971; DeWalt et al. 2013). The sole state record is from Fort Ancient State Memorial in southwestern Ohio (Figure 7).

#### *Prostoia completa* (Walker)

**Collection records. USA, Ohio:** Richland Co., Opossum Run, Hwy 95 near junction with Clear Fork Mohican River, 40.6274, 82.3880, 22.IV.1989, RW Baumann and SM Clark, ♂, ♀ (BYU); Scioto Co., Pond Lick Creek, 4 km NW Friendship, SSF, 38.7064, 83.1378, 15.IV.2006, RE DeWalt and SK Ferguson, 2♂ (INHS).

**Remarks.** This species is distributed very broadly across the eastern Nearctic region (DeWalt et al. 2013). It is known in Ohio from only 2 localities in the south-central portion of the state (Figure 8), but several additional populations across the state are expected to be located. This species typically inhabits large streams and small rivers, whereas *P. similis* is found typically in smaller upland systems.

#### *Neoperla coosa* Stark and Smith

**Collection records. USA, Ohio:** Clermont Co., Little Miami River, Batavia at OH 222 bridge over Backbone Creek, 39.0867, 84.1798, 18.VII.1996, no collector information, ♂ (TNHS); East Fork Little Miami River, Binning Rd., E SR 222, 39.1190, 84.2089, 15.VIII.1995, Trybula, 3♂ (OBS); same but 5.VI.1997, no collector information, ♂ (OBS); Backbone Creek, SR 222 near confluence with Little Miami River, 39.0866, 84,1767, 27.VII.1995, Trybula, 5♀ (OBS); same but 15.VIII.1995, Trybula, ♂ (OBS); Lake Co., Grand River, 3.5 km SSW Madison, Hidden Valley Park, 41.7424, -81.0506, 26.VI.2006, RE DeWalt, 8♂, 31♀ (INHS).

**Remarks.** Until recently, *N. coosa* was known only from Alabama, Indiana, North Carolina, and Tennessee (Stark 2004; DeWalt et al. 2013). Given that Myers et al. (2011) recently reported this species from the Adirondack Mountain region of northern New York, and given that this species is superficially very similar to *N. clymene* (Stark 2004), it likely has a much broader distribution across the eastern Nearctic region. There are 4 locality records for Ohio (Figure 8), 1 from the Grand River in the far northeastern portion, and 3 from the Little Miami River catchment in Clermont County. More Ohio records are likely to accrue with further collecting efforts.

#### *Perlinella drymo* (Newman)

**Collection records. USA, Ohio:** Athens Co., Hocking River, Athens, 39.3292, -82.1222, 5.V.1932, WC Stehr, ♀ (INHS); Butler Co., Indian Creek, 3.5 km NE Ross at Hwy 128, 39.3374, -84.6272, 10.III.1953, AR Gaufin, 3♂, 1♀, 6 nymphs (BYU); Lorain Co., [Plum Creek], 41.2939, -82.2174, 4.IV.1891, no col-lector information, ♂ (OHS); Warren Co., Little Miami River, Morrow, 39.3571, -84.1286, 14.III.2007, RE DeWalt, nymph (INHS).

**Remarks.** This species exhibits a very broad distribution across the eastern Nearctic region (Stark 2004; DeWalt et al. 2013). This species typically occurs in large streams and riverine systems. Although there are only 4 known records for Ohio (Figure 8), more are likely to accrue with contemporaneous collecting.

#### *Isoperla dicala* Frison

**Collection records. USA, Ohio:** Columbiana Co., tributary to North Fork Little Beaver River, west slope rivulet descending Pancake Rd., 40.7530, -80.5409, 18.II.1978, nymph (Tkac 1979).

**Remarks.** This species is very broadly distributed across the central and eastern Nearctic regions (DeWalt et al. 2013). The only Ohio record is based on a single nymph collected from a small upland tributary in the far eastern rim of the state (Figure 8).

#### *Isoperla signata* (Banks)

**Collection records. USA, Ohio:** Geauga Co., Chagrin River, 3 mi S Chesterfield at OH 306, 41.4900, -81.3402, 14.III.1990, RW Baumann and RF Kirchner, nymph (BYU); Chagrin River, 2 km N South Russell, Taber Reserve, 41.4507, -81.3701, 27.I.2007, RE DeWalt and J Keiper, 4 nymphs (INHS); Portage Co., Tinkers Creek State Park, 41.2850, -81.3944, 1.V.1978, 3 nymphs (Tkac 1979); tributary to Aurora Branch Chagrin River, 4 km N Aurora at OH 306, 41.3439, -81.3421, 15.III.2005, RE DeWalt, 4 nymphs (INHS).

**Remarks.** This species is distributed in the more northern parts of central and eastern Nearctic states and Canadian provinces (DeWalt et al. 2013). Within Ohio, there are 4 valid records from the Chagrin and Ashtabula River basins in the far northeastern portion of the state (Figure 9).

### Nearctic—widespread

#### *Nemoura trispinosa* Claassen

**Collection records. USA, Ohio:** Brown Co., East Fork Little Miami River, Hwy 50, 39.1866, -83.9374, 3.V.1952, AR Gaufin, 8♂, 6♀ (BYU); Champaign Co., Mosquito Creek, 3 km NW Millerstown, Kiser Lake Wetland State Nature Preserve (KLWSNP), 40.2027, 83.9875, 6.VI.1996, RA Vargo, ♂ (OBS); KLWSNP, 40.1867, -83.9545, 29.IV.1999, M Gray, 2 nymphs (OEPA); Clark Co., Rock Run, Springfield, 39.9269, -83.8703, 30.V.1953, AR Gaufin, ♂ (BYU); Greene Co., Spring Glen, Yellow Springs, 39.8003, 83.8838, 7.VI.1953, AR Gaufin, 17♂, 20♀ (BYU); headwaters of spring joining Little Miami River, 2 mi S Yellow Springs, 39.7803, -83.9036, 20.IV.1989; RW Baumann and RF Kirchner, ♀, 4 nymphs (BYU); spring stream, JBSP, 39.7864, -83.8637, 20.IV.1989, RW Baumann and RF Kirchner, 3♂, 4♀ (BYU); same but 20.IV.1989, RW Baumann and SM Clark, 1 nymph (BYU); same but 7.VI.1989, RW Baumann and SM Clark, 6♂, 3♀ (BYU); Portage Co., Shalerville spring, along Cuyahoga River, 300 m S of Rt. 303, 41.2433, -81.2893, 14.V.1998, JB Keiper, ♀ (CMNH); same but 26.V.1998, PL Brutsche, 3♂, ♀ (CMNH); same but 2.VI.1998, PL Brutsche, 4♂, 2♀ (CMNH); same but 8.XII.1998, JB Keiper, ♀ (CMNH); Wayne Co., stream at Newkirk Church, Clinton Township, 40.6789, -82.0997, 29.VI.1967, JA Beatty, 14 nymphs (INHS).

**Remarks.** This species is distributed broadly from the tundra-boreal forest interface south throughout much of the once-glaciated landscapes (DeWalt et al. 2013). Within the state, *N. trispinosa* species has been collected from several widely-disjunct localities (Figure 9), including series collected as recently as the late 1990s from springs in eastern Ohio. This is a glacial relict and likely the southern-most known extant population of this species. The closest known recent records for *N. trispinosa* are from northern Pennsylvania (Earle 2009) and southwestern Michigan (Allegan Co., Silver Creek at springhead, 7 km SE Hamilton, Allegan State Game Area, 42.6529, -85.9197). This species has not been collected from adjacent Indiana (Grubbs 2004; DeWalt and Grubbs 2011).

#### *Taeniopteryx parvula* Banks

**Collection records. USA, Ohio:** Adams Co., Ohio Brush Creek, Hwy 73 nr. Serpent Mound State Memorial, 39.0228, -83.4358, 26.II.2005, MH Alford, ♂ (BPSC); Ashtabula Co., Phelps Creek, S Windsor Rd., 41.5090, 80.9274, 18.II.1978, ♂ (Tkac 1979); same but 3.III.1979, 2♂, 3♀ (Tkac 1979); Franklin Co., Scioto River, Columbus, 39.9611, -82.9989, 5.III.1938, TH Frison, ♀ (INHS); Lake Co., Paine Creek, Seeley Rd., Leroy Township, Indian Point Memorial Park, 41.7170, 81.1708, 8.III.1976, ♀ (Tkac 1979); Warren Co., Todds Fork, Morrow, 39.3451, -84.1120, 27.II.1952, AR Gaufin, ♀ (BYU).

**Remarks.** This species is broadly distributed across the Nearctic region, extending as far west as Alberta, Colorado, and New Mexico (Kondratieff and Baumann 1988; Stewart 2000; DeWalt et al. 2013). East of the Mississippi River, this species is markedly more common in once-glaciated landscapes, and there is strong evidence for statewide disappearance in Illinois and Indiana (DeWalt et al. 2005; DeWalt and Grubbs 2011). The only records for Ohio are from 5 widely-dispersed localities ranging from Ashtabula County south to Adams County (Figure 9).

#### *Pteronarcys dorsata* (Say)

**Collection records. USA, Ohio:** Franklin Co., Columbus, 39.9610, -82.9990, 1.V.1906, GB Merrell, ♀ (INHS).

**Remarks.** This species exhibits a very broad distribution pattern across the Nearctic region (Nelson 2000; DeWalt et al. 2013) and typically inhabits larger streams and riverine systems (e.g., Barton 1980; Lechleitner and Kondratieff 1983). There is only 1 valid state record (Figure 9), but there were several other records of nymphs without lateral abdominal projections. It is highly likely that *P. pictetii* (Newman) will be found in Ohio, especially when many nymphs have been reared to the adult stage. Adults provide the only reliably identified life stage for *Pteronarcys*.

### Summary

Plecoptera in Ohio have been heavily collected since the 1940s. Two regions of Ohio, however, may have been undersampled (Figure 1). Northwestern Ohio has been lightly collected, but its low diversity is a reflection of its original, relatively-depauperate fauna, as most of this area was composed of flat lake plains with extensive marshes prior to human settlement (DeWalt et al. 2012). This region is now largely agricultural with extreme hydrological modification due to channelization of streams and the installation of networks of underground tiles.

Although the greater Hocking Hills area seems to have been sufficiently sampled, new species locality records (e.g., *L. tenella*) continue to accrue. Of greater need for continued collecting efforts is the area south of Hocking Hills (e.g., the western portion of Wayne National Forest and Shawnee State Forest) and extending northeastward towards West Virginia's northern panhandle (i.e., southeastern Ohio). Much of this area is nested within the Western Allegheny Plateau and likely holds several additional locality records for species with Appalachian affinities.

Many species of Ohio Plecoptera should be considered for endangered or threatened status. The 4 perlid species no longer present in the state, plus the 3 additional perlids species that have experienced sharp range reductions, share life history traits with long nymphal growth periods and lack an egg diapause (DeWalt et al. 2005). The combination of these traits, plus a slow accrual of watershed-scale disturbance of riverine systems, has likely led to the loss or near-loss of these species from Ohio waters (DeWalt et al. 2012). The next group of species in likely peril in Ohio are those that are known only from the cooler, northeastern portion of the state (e.g., *A. pechumani, A. concolor/neglecta, M. iroquois, P. biloba*). Distribution modeling using Ohio and regional occurrence data is underway and will aid in assessing vulnerability of the fauna as a whole to range loss and subsequent disappearance both within the state and the broader Midwestern region.

**Table 1.**
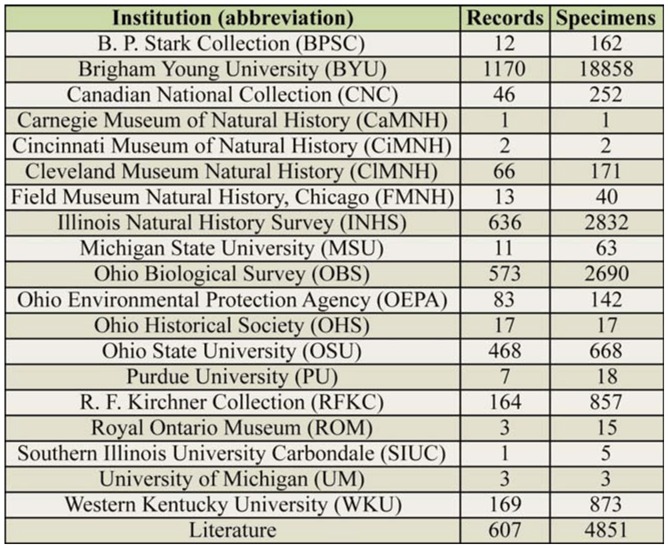
Institution/collection, number of stonefly specimen records, and number of specimens examined.

**Table 2.**
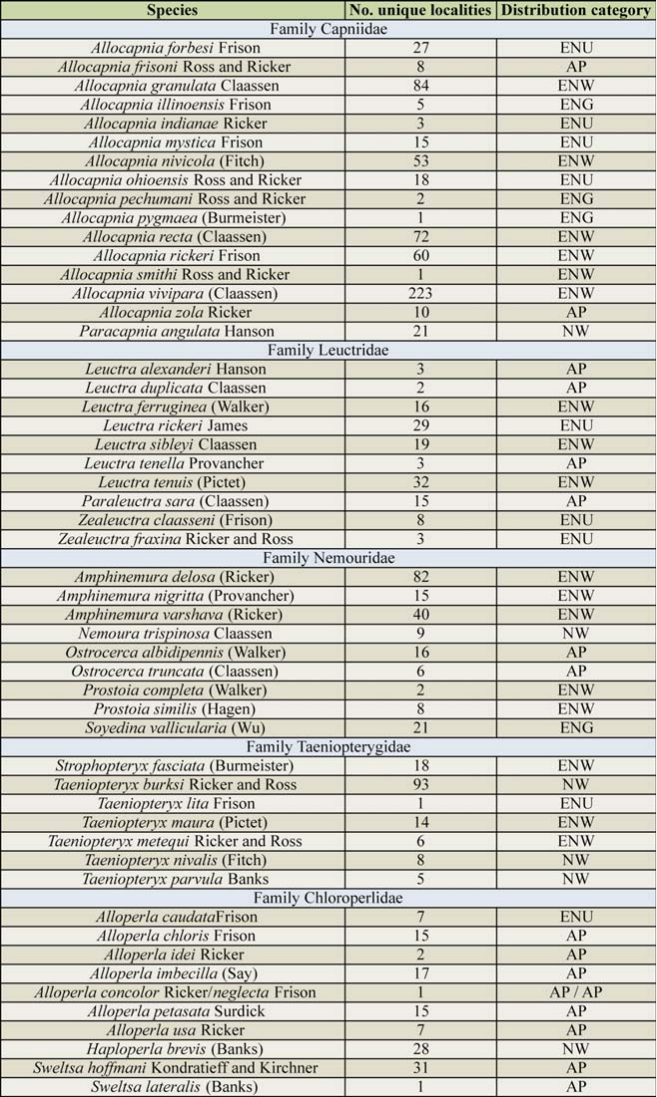
List of the stonefly species reported from Ohio, with number of unique collection localities and Nearctic-scale distribution categories. Families are arranged phylogenetically according to DeWalt et al. (2013).

**Table 2.**
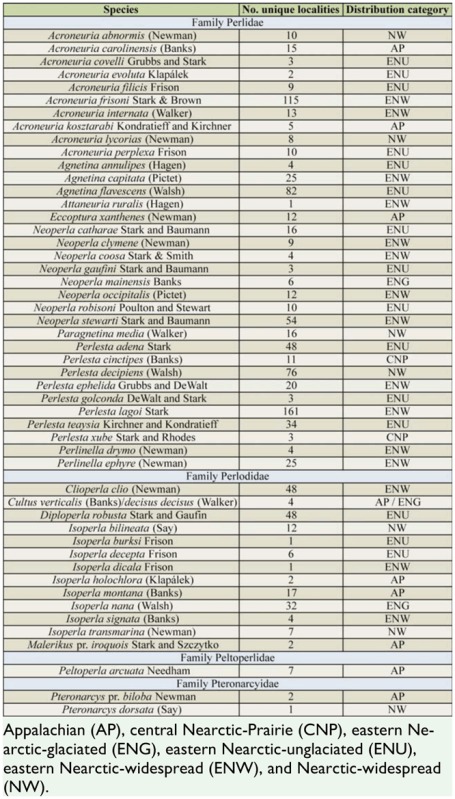
Continued.
